# A Deep Q-Network-Based Algorithm for Multi-Connectivity Optimization in Heterogeneous Cellular-Networks †

**DOI:** 10.3390/s22166179

**Published:** 2022-08-18

**Authors:** Juan Jesús Hernández-Carlón, Jordi Pérez-Romero, Oriol Sallent, Irene Vilà, Ferran Casadevall

**Affiliations:** Signal Theory and Communications Department, Universitat Politècnica de Catalunya (UPC), 08034 Barcelona, Spain

**Keywords:** multi-connectivity, deep learning, Deep Q-Network, heterogeneous networks, cellular networks, 5G NR, LTE, O-RAN

## Abstract

The use of multi-connectivity has become a useful tool to manage the traffic in heterogeneous cellular network deployments, since it allows a device to be simultaneously connected to multiple cells. The proper exploitation of this technique requires to adequately configure the traffic sent through each cell depending on the experienced conditions. This motivates this work, which tackles the problem of how to optimally split the traffic among the cells when the multi-connectivity feature is used. To this end, the paper proposes the use of a deep reinforcement learning solution based on a Deep Q-Network (DQN) in order to determine the amount of traffic of a device that needs to be delivered through each cell, making the decision as a function of the current traffic and radio conditions. The obtained results show a near-optimal performance of the DQN-based solution with an average difference of only 3.9% in terms of reward with respect to the optimum strategy. Moreover, the solution clearly outperforms a reference scheme based on Signal to Interference Noise Ratio (SINR) with differences of up to 50% in terms of reward and up to 166% in terms of throughput for certain situations. Overall, the presented results show the promising performance of the DQN-based approach that establishes a basis for further research in the topic of multi-connectivity and for the application of this type of techniques in other problems of the radio access network.

## 1. Introduction

With the ongoing increase of data traffic as well as the emergence of new services with high speed and reliability requirements, Mobile Network Operators (MNO) are actively trialing and deploying fifth generation (5G) networks in an effort to support new vertical-driven use cases and enhanced user experiences [1]. 5G New Radio (5G NR) deployments are progressively introduced on top of existing legacy technologies, such as Long-Term Evolution (LTE), leading to heterogeneous deployments with multiple radio access technologies (RATs), different cell types, such as macrocells, indoor and outdoor small cells, and operating in a wide range of spectrum bands (e.g., sub-6 GHz bands used by all RATs and millimeter wave (mmW) bands used by 5G New Radio). Network densification has been a mainstay of LTE networks, and the need for small cells will be even more critical in 5G and beyond systems due to operation in higher spectrum bands and the need to support traffic densities that are two to three orders of magnitude higher than in LTE [2]. The general industry consensus is that 5G will drive hyperdense deployments with site densities in excess of 150 sites/km^2^ in urban and selected indoor scenarios [3].

In this context, multi-connectivity (MC) has emerged as a key technology for supporting simultaneous access via LTE and 5G networks [4], and is also expected to play a key role in further network evolutions towards the sixth generation (6G). The central idea behind the MC concept is that User Equipment (UE) has connectivity with different nodes of the Radio Access Network (RAN) at the same time, e.g., eNodeBs (eNB) operating with LTE and/or gNodeBs (gNB) operating with 5G NR [5]. There is one master node (MN) responsible for the radio-access control plane and one, or in the general case multiple, secondary node(s) (SN), that provide additional user-plane links. In this way, the UE can be provided with radio resources from distinct eNBs/gNBs, which has benefits in terms of additional capacity for better supporting the data rate and reliability requirements of 5G and beyond systems. At the same time, this brings the challenge of properly managing the MC operation through the configuration of the traffic sent through each node. This allows exploiting those situations where MC is more beneficial in front of other situations in which MC may not be the best option depending on the load in the involved nodes or the propagation conditions experienced by the UE.

In the Third Generation Partnership Project (3GPP), MC is specified through the Multi-Radio Dual Connectivity (MR-DC) feature defined in [6] that considers different options depending on the technology used by the MN and by the SN, and on the core network technology (i.e., 5G core or Evolved Packet Core (EPC)). The operation of MR-DC is built upon the use of three different types of radio bearers [6], namely the Master Cell Group (MCG) bearers in which data is transmitted through the MN, the Secondary Cell Group (SCG) bearers in which data is transmitted through the SN, and the Split bearers, in which data is split between the SN and the MN at the Packet Data Convergence Protocol (PDCP) layer of the radio interface protocol stack. The MR-DC feature between LTE and 5G is widely used nowadays in the current 5G Non-StandAlone (NSA) deployments that have enabled a quick introduction of 5G NR making use of the EPC core network of the legacy LTE systems [5].

The interest in MC is also reflected in some 3GPP Release 17 study items that have addressed the MR-DC with multiple cells operating in different bands. One example is [7], which considers up to four bands in LTE and two bands in 5G NR, one for the sub-6GHz Frequency Range 1 (FR1) and the other for the mmWave Frequency Range 2 (FR2). Moreover, several works have also recently considered the use of MC in contexts that are relevant for future beyond 5G and 6G systems, such as the communication over mmWave and THz frequency bands [8], the multi-connectivity between a base station and other UEs acting as relays [9], and hybrid satellite and terrestrial networks [10], in which for example the joint use of a reconfigurable intelligent surface and a base station are used to strengthen satellite signals in [11].

From an algorithmic perspective, the literature has considered different problems in relation to MC, such as resource allocation [12,13], cell and RAT selection [14,15], and traffic split [16,17,18,19]. Concerning resource allocation, a Smart Aggregated RAT Access (SARA) strategy is proposed in [12] for joint RAT selection and resource allocation in a scenario with cellular base stations and Wi-Fi access points. The solution makes use of a Semi Markov Decision Process (SMDP)-based hierarchical decision framework (HDF). In [13] the optimization problem of resource allocation in a MC scenario with 5G and LTE is formulated. Then, a solution based on two heuristic algorithms is proposed, namely a base station selection algorithm performed by the UE and a resource block allocation algorithm executed by the base station.

The problem of secondary base station selection in MC with 5G/LTE is addressed in [14], which presents different algorithms aimed at improving robustness and performance while minimizing the energy consumption. In turn, [15] considers an LTE/WiFi scenario and proposes a network switching strategy based on a Markov Decision Process and a value iteration algorithm to determine the set of RATs that a user is connected to in each handover window.

The problem of traffic split between different RATs is considered in [16] focusing on an LTE/Wi-Fi scenario. The paper assumes a heterogeneous network controlled by a single coordinating node that collects relevant information, decides on the best choice of RAT for all users, and advises on the actual amount of radio resources that every user may utilize on each technology. The problem is formulated analytically, and a solution based on the weighted max-min algorithm is proposed. In [17] the problem of traffic split between the master and the secondary eNB in LTE with dual connectivity is modelled as a Constrained Markov Decision Process, and a solution based on the Lagrangian approach is proposed. Similarly, [18] considers a scenario with 5G-LTE multi-connectivity, and makes use of Lagrangian Dual Decomposition to determine the fraction of traffic transmitted through each cell that maximizes the goodput, whereas [19] formulates a PDCP split bearer decision problem that decides whether and how to split the traffic across multiple cells in order to meet the bandwidth requirements of user services and proposes a heuristic solution to solve the problem.

This paper addresses the traffic split multi-connectivity problem in multi-RAT scenarios. The target is to determine a policy to optimally distribute the traffic of a UE across the different RATs and cells by fulfilling the QoS requirements while minimizing the resource consumption of the UE and ensuring that no overload situations arise in the involved cells.

The novelty of the paper with respect to prior works dealing with traffic split [16,17,18,19] is the use of Deep Reinforcement Learning (DRL), and in particular the Deep Q-Network (DQN) technique [20], in order to learn the traffic split policy to be applied on a per UE basis so that the amount of bandwidth used by a UE is minimized while at the same time providing the required bit rate and avoiding overload situations in the involved cells. To the authors’ best knowledge, the use of DQN has not been considered by other researchers in the context of traffic split for MC. Instead, previous works have considered different algorithmic solutions and different optimization targets, such as linear programming for ensuring fairness in [16], Lagrangian multipliers for minimizing delay in [17], Lagrangian Dual Decomposition for optimizing goodput in [18] or a heuristic algorithm for maximizing the number of served UEs in [19]. Following the trend of applying machine learning for different problems in the RAN [21] and for different applications [22], the motivation to consider DRL in MC is that DRL techniques are useful for optimizing dynamic decision-making problems in the absence of an accurate mathematical model of the operational environment. Moreover, thanks to their capability of generalizing from past experience, DRL techniques are efficient in problems that depend on a large number of input variables and in which both the inputs and the decision-making outputs can take a large range of possible values, as it is the case of the MC problem considered here.

Based on a first version of the DQN model presented in our recent work [23] and further evolved in [24], this paper significantly extends these works by presenting the detailed formulation and algorithmic solution of the DQN model, the architectural framework based on the open RAN (O-RAN) architecture [25] for supporting the proposed solution, and by providing a much more exhaustive performance assessment of the algorithm considering different evaluation conditions. The O-RAN based framework for supporting the implementation of the proposed approach constitutes another novelty of the paper with respect to previous works.

The rest of the paper is organized as follows. Section 2 presents the system model, formulates the considered multi-connectivity problem and presents the O-RAN-based architectural framework. Section 3 presents the proposed DQN-based solution. Different performance results are provided in Section 4. Finally, Section 5 summarises the conclusions.

## 2. Multi-Connectivity Model Formulation

### 2.1. Problem Definition

Let us consider a heterogeneous RAN where different UEs with multi-connectivity capabilities are camping. A given *u*-th UE considers *M* different RATs and *N* different cells per RAT as candidates for the multi-connectivity. Then, let us denote as *A_u_* = {*C_m,n_*} the set of candidate cells detected by the *u*-th UE, where *C_m,n_* denotes the *n*-th cell of the *m*-th RAT with *n* = 1, …, *N* and *m* = 1, …, *M.* It is worth mentioning that, due to the mobility of the UE, the specific detected cells in a given RAT may change with time. In this respect, it is assumed that the *N* candidate cells of a RAT correspond to the best *N* cells detected by the UE at a certain time based on measurements averaged during a time window of duration Δ*T* s.

Through the use of multi-connectivity, the traffic of the *u*-th UE is split across multiple RATs/cells of the set *A_u_*. It is assumed that, at a certain time, the UE can be simultaneously connected to a maximum of *N_max_* cells among the *M*·*N* candidates. The multi-connectivity configuration of the *u*-th UE can be expressed as the *M* × *N* matrix **B_u_** = {*β_m,n_*}, where *β_m,n_* ∈ [0, 1] defines the fraction of total traffic of the *u*-th UE that is delivered through the *n*-th cell of the *m*-th RAT. Then, the objective is to find the optimal configuration **B_u_** = {*β_m,n_*} to be applied in a time window of Δ*T* that allows ensuring the Quality of Service (QoS) requirements with minimum resource consumption and avoiding overload situations in the different RATs/cells. In this respect, it is assumed that the QoS requirement of the user *u* is expressed in terms of a required bit rate *R_u_* (b/s) to be provided.

To formalize the problem, let us denote as *T_u_*(**B_u_**) the total throughput or bit rate obtained by user *u* during the last time window period Δ*T* as a result of the multi-connectivity configuration **B_u_**. Let us also denote *a_m,n_*(*β_m,n_*) as the number of physical resources in the *m*-th cell and *n*-th RAT assigned to the *u*-th UE to transmit the traffic corresponding to fraction *β_m,n_*. Assuming LTE and 5G NR-based RATs, the physical resources correspond to the Physical Resource Blocks (PRBs), each one defined as a set of 12 contiguous subcarriers in an OFDMA access [3]. Then, considering that *b_m,n_* is the bandwidth of one PRB in the *m*-th cell and *n*-th RAT, the bandwidth allocated to the user *u* in this RAT, denoted as $\gamma \left(\beta_{m,n}\right)$, is given by:

(1) $$\gamma \left(\beta_{m,n}\right)=a_{m,n}\left(\beta_{m,n}\right)\cdot b_{m,n}$$

In addition, the total fraction of occupied PRBs in a RAT/cell accounting for all the UEs connected to that cell is denoted as *ρ_m,n_*(*β_m,n_*).

With all the above, the considered problem to be solved for the *u*-th UE is formally defined as:

(2) $$B_{u}=\underset{Bu}{arg\;min}\left[\frac{1}{w_{max}}\sum_{m=1}^{M}\sum_{n=1}^{N}\gamma \left(\beta_{m,n}\right)\right]\;s.t.\;T_{u}(B_{u})\geq R_{u}\;,\;\;\rho_{m,n}\left(\beta_{m,n}\right)\leq \rho_{max}\;\forall m,n\;\overset{M}{\underset{m=1}{\sum }}\overset{N}{\underset{n=1}{\sum }}\beta_{m,n}=1$$

where $w_{max}$ is the maximum possible bandwidth to be assigned to the user *u* and *ρ_max_* ∈ [0, 1] is the maximum threshold established to avoid overload situations in a cell. Then, the problem in Equation (2) intends to find the multi-connectivity configuration **B_u_** that minimizes the fraction of total bandwidth allocated to the *u*-th UE while at the same time ensuring that the provided throughput is above the required bit rate, i.e.,$\;T_{u}$(**B_u_**)$\;\geq R_{u}$ and that the total fraction of occupied PRBs in the involved RATs/cells is lower than a threshold $\rho_{max}$. It is worth mentioning that in the problem of Equation (2) the effects of mobility, propagation and interference are implicitly captured in the term $\gamma \left(\beta_{m,n}\right)$. Specifically, as the UE moves, its propagation and interference conditions with respect to the different cells changes and, therefore, the involved cells modify the amount of bandwidth to be allocated to the UE so that the traffic fraction *β_m,n_* can be transmitted.

### 2.2. Proposed O-RAN Based System Architecture for MC Configuration

**Figure 1:**
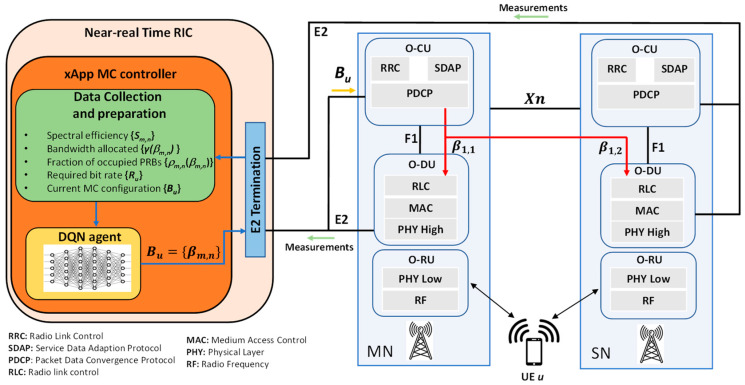
O-RAN-based architectural components for supporting the MC configuration.

In order to enforce in the network, the multi-connectivity configuration **B_u_** obtained as a result of the above problem, this paper proposes the system architecture depicted in Figure 1. It is based on the O-RAN Alliance reference architecture [25], which complements 3GPP 5G standards with a foundation of virtualized network elements, white-box hardware and standardized interfaces that fully embrace the core principles of openness and intelligence. One of the principal characteristics of O-RAN architecture is the RAN disaggregation, which splits a 5G NR base station (gNB) into different functional units, namely a Central Unit (CU), a Distributed Unit (DU), and a Radio Unit (RU) (called O-CU, O-DU, and O-RU in O-RAN specifications). The O-CU can be further split into two logical components, one for the Control Plane (CP), and one for the User Plane (UP). The O-RAN architecture also considers the use of LTE technology with so-called O-eNB nodes. The O-CU hosts the upper layers of the radio interface protocol stack. These include the PDCP layer that splits the traffic in case of multi-connectivity, in addition to the Radio Resource Control (RRC) and the Service Data Adaptation Protocol (SDAP) layers for control plane and user plane, respectively, that are on top of PDCP. In turn the O-DU hosts the lower layers of the protocol stack, namely the Radio Link Control (RLC), the Medium Access Control (MAC), which hosts the scheduler in charge of allocating the PRBs to the different UEs, and the upper parts of the Physical (PHY) layer (e.g., channel coding, modulation). Finally, the O-RU hosts the lower parts of the PHY layer (e.g., Inverse Fast Fourier Transformation (IFFT) for Orthogonal Frequency Division Multiple Access (OFDMA) transmission) and the Radio Frequency (RF) functions.

The multi-connectivity situation illustrated in Figure 1 considers the downlink traffic transmitted to a UE served by two cells of 5G NR RAT *m* = 1. The cell *n* = 1 is at the MN while the cell *n* = 2 is at the SN. The multi-connectivity configuration for the different users is determined by an MC controller, which can be hosted at the near-real time RAN Intelligent Controller (near-RT RIC) of the O-RAN architecture. The near-RT RIC is deployed at the edge of the network, is able to operate control loops with a periodicity between 10 ms and 1 s, and it can interact with the DUs and CUs in the RAN. A relevant characteristic of the near-RT RIC is that it supports the execution of third-party applications, referred to as xApps [26]. Then, the MC controller can be implemented as one of these xApps.

The inputs of the MC controller are different measurements (further explained in next section) from the RAT/cells, as shown in Figure 1. These measurements can be sent to the near-RT RIC through the E2 interface. At the near-RT RIC, they are sent to the xApp MC controller where they are collected and processed in order to be adapted to the format required by the DQN agent that constitutes the core of the MC controller, as is explained in Section 3. The output of the MC controller is the configuration **B_u_** = {*β_m,n_*} with the weights *β_m,n_* to be configured at the PDCP layer of the MN, which resides in the O-CU. This configuration can be established via the E2 interface that supports enabling, disabling or modifying the dual connectivity process [27]. Based on this configuration, the PDCP splits the traffic between the MN and the SN, and delivers the part of the traffic of the SN via the Xn interface that interconnects the MN and the SN. Then, the MAC scheduler at each O-DU allocates the necessary amount of bandwidth resources $\;\gamma \left(\beta_{m,n}\right)$ to the UE to transmit the fraction of traffic *β_m,n_* corresponding to the cell. The specific design of the MAC scheduler is out of the scope of this work, but in general it considers aspects such as the instantaneous propagation and interference conditions observed by the UE, the QoS requirements, the amount of UEs in the cell, etc.

## 3. DQN-Based Solution

To address the problem defined by (2) a large number of variables must be considered, such as the propagation and interference conditions experienced by the UE in the links with the different cells/RATs, the existing load in each cell, and the QoS requirements, among others. Additionally, there is also a dependence of the behavior of the MAC layer in each cell/RAT that determines the amount of resources allocated to the UE. However, since the MC controller operates on top of the different RATs, in general does not have a precise model of how these resource allocation techniques work to determine the value of $\gamma \left(\beta_{m,n}\right)$ and its impact on the QoS metrics. Then, given the complexity and the multiplicity of inputs to the multi-connectivity problem, DRL techniques are considered as solid candidates for approaching it. Particularly, this paper relies on the DQN algorithm, which is a model-free and value-based DRL algorithm that considers discrete action spaces. This algorithm has been already successfully applied to other problems in the RAN, such as capacity sharing in [28], resource allocation in heterogeneous networks in [29], and spectrum sharing in 4G/5G networks in [30].

Other more sophisticated techniques, such as Double DQN (DDQN) or Deep Deterministic Policy Gradients (DDPG), could be considered to overcome the overestimation of the Q values in DQN. A previous work of the authors in the area of capacity sharing [31], where DQN was compared against DDQN and DDPG, suggested retaining DQN for this work. While no significant differences in terms of performance would be expected, practicality considerations such as the speed of the training process, and the number of hyperparameters to configure, may favor DQN.

In the proposed approach, learning is a dynamic process carried out by the DQN agent located at the MC controller, which makes decisions on the multi-connectivity configurations for the different UEs. The agent operates in discrete times with granularity equal to the time window duration Δ*T*. These discrete times are denoted as *t*, *t* + 1, …, *t* + *k*, … At time *t*, the DQN selects an action ***a(t)*** that contains the MC configuration to be applied for a given UE in the next time window. The action selection is based on the current state at time *t*, denoted as ***s*(*t*)**, and on the decision-making policy available at this time. Then, as a result of applying the selected MC configuration, a reward signal *r*(*t* + 1) is provided to the DQN agent at the end of the time window. This reward signal measures how good or bad was the last performed action ***a(t)*** according to the considered optimization criteria and, in consequence, this obtained signal is used to improve the decision-making policy. The different components of this process are detailed in the following.

### 3.1. State, Action and Reward Specification

The state ***s(t)*** is a vector that includes the following components for a given *u*-th UE:

- Requirement of the *u*-th UE: *R_u._*
- Spectral efficiency per RAT/cell {*S_m,n_*} of the *u*-th UE.
- Current configuration **B_u_** = {*β_m,n_*}, which corresponds to the configuration applied at time *t*-1 to the *u*-th UE.
- Fraction of occupied bandwidth resources by the *u*-th UE in each RAT/cell {$\gamma \left(\beta_{m,n}\right)$}.
- Fraction of total occupied bandwidth resources in each RAT/cell {*ρ_m,n_*(*β_m,n_*)}.

All the values *S_m,n_*,$\;\gamma \left(\beta_{m,n}\right)$ and *ρ_m,n_*(*β_m,n_*) are average values measured during the last time window of duration Δ*T*, i.e., between discrete times *t*-1 and *t*. Notice that the state has a total of 1 + 4·*N*·*M* components.

Each action ***a(t)*∈** A represents a matrix **B_u_** = {*β_m,n_*} that corresponds to the MC configuration to be applied during the next time window Δ*T*. The action space A includes all the possible MC configurations, and is defined considering that the possible *β_m,n_* values are discretized with granularity Δ*β* and that the aggregate of all *β_m,n_* values in matrix **B_u_** equals 1. Moreover, the action space also considers that a UE can be connected to a maximum of *N_max_* cells. Therefore, at most *N_max_* values of *β_m,n_* can be different from 0 in a certain action.

The reward *r*(*t* + 1) measures how good or bad was the performance obtained by the last action ***a(t)*** for the state ***s(t)*** in relation to the target and constraints of the optimization. Then, considering the optimization problem (2), and that the last action ***a(t)*** is given by MC configuration **B_u_** = {*β_m,n_*}, the reward is defined as:

(3) $$\;\;\;\;\;\;\;\;\;\;r\left(t+1\right)=\left(1-\frac{1}{w_{max}}\overset{M}{\underset{m=1}{\sum }}\overset{N}{\underset{n=1}{\sum }}\gamma \left(\beta_{m,n}\right)\right)\cdot min\left(1,\frac{T_{u}\left(B_{u}\right)}{R_{u}}\right)\cdot \underset{\begin{array}{c} m,n\\ \beta_{m,n}>0 \end{array}}{\prod }min\left(1,\frac{\rho_{max}}{\rho_{m,n}\left(\beta_{m,n}\right)}\right)$$

The first multiplicative term in *r*(*t* + 1) captures the total bandwidth assigned to the *u*-th UE in all the cells/RATs, so the lower the amount of bandwidth assigned the higher the reward, and this reflects a better fulfilment of the optimization target in Equation (2). The second term multiplicative represents a penalty introduced when the achieved throughput *T_u_*(**B_u_**) is lower than the minimum requirement *R_u_*, corresponding to the first constraint in Equation (2). The last multiplicative term introduces a penalty for each cell/RAT in which the UE has transmitted traffic (i.e., *β_m,n_* > 0) and the cell is overloaded, thus capturing the second constraint in Equation (2). Note that the values of $\gamma \left(\beta_{m,n}\right)$, *ρ_m,n_*(*β_m,n_*) and *T_u_*(**B_u_**) correspond to the averages obtained during the time window Δ*T* between discrete times *t* and *t* + 1.

### 3.2. Policy Learning Process

The training process is used to dynamically learn the decision-making policy π that the DQN agent uses when selecting the different actions as a function of the current state. For this purpose, the DQN agent executes the DQN algorithm of [16] but particularized to the state, action and reward signals presented above.

In general, reinforcement learning (RL) algorithms aim at finding the optimal policy π* that maximizes the discounted cumulative future reward (i.e., $\overset{\infty }{\underset{j=0}{\sum }}\tau^{k}r(t+j+1)$, where τ is the discount rate ranging *0 ≤* τ *≤* 1). In value-based RL algorithms, such as DQN, this is done by obtaining the optimum action-value function Q*(***s,a***), which is a scalar value representing the maximum expected discounted cumulative reward starting at time *t* from state ***s***, taking the action ***a*** and following the policy *π*. This can be expressed in a recursive form by the Bellman equation as:

(4) $$Q^{*}\left(s,a\right)=E[r\left(t+1\right)+\tau \cdot \underset{a^{\prime }}{max}Q^{*}\left(s\left(t+1\right),a^{\prime }\right)|s\left(t\right)=s,a\left(t\right)=a,\;\pi ]$$

Given Q*(***s,a***), the optimum policy is defined by greedily selecting the action ***a*** with the highest value for each state ***s***, that is:

(5) $$\pi^{*}=\underset{a}{\arg max}Q^{*}\left(s,a\right)$$

To determine the optimum action-value function, DQN approximates Q*(***s,a***) by a deep neural network (DNN) with weights *θ,* denoted as *Q*(***s,a***, *θ*), which is progressively updated during the training process. This DNN takes as input the state ***s*** and provides as output the value for each possible action ***a*** in accordance with the weights *θ*, which define the interconnections between the different neurons. For updating *Q*(***s,a***,*θ*), the DQN agent is composed of the following elements:

- Evaluation DNN *Q*(***s,a***,*θ*): this is the main approximation of the optimum action-value function Q*(***s,a***). It is used to determine the decision-making policy *π* for selecting the actions, as:
  (6) $$\pi =\underset{a}{argmax}\;Q\left(s,a,\theta \right)$$
- Target DNN *Q*(***s,a***, *θ^-^*): this is another DNN with the same structure as the evaluation DNN but with weights *θ^-^*. It is used to obtain the Time Difference (TD) target $r(t+1)+\tau \underset{a^{\prime }}{\;max}\;Q\left(s(t+1),a^{\prime },\;\theta^{-}\right)$ that is used for making successive updates of the evaluation DNN during the training. Moreover, this DNN is updated every *P* time steps (i.e., time windows) with the weights of the evaluation DNN, i.e., *θ^-^* = *θ*.
- Experience dataset *D*: this is a dataset of length *l* that stores the experiences of the DQN agent. The stored experience at time *t* is represented by the tuple < ***s***(*t*), ***a(t)***, *r*(*t* + 1), ***s***(*t* + 1) >, which captures the state at *t*, the action taken, and the resulting reward and new state at time *t* + 1. The stored experiences are randomly selected during the training process to update the weights *θ.*

At initialization, the weights of both the evaluation and target DNNs are randomly selected. Then, they are updated as a result of the training process of the DQN agent, which is divided in two parts: the data collection and the update of weights *θ*.

Data collection consists in gathering experiences and storing them in the experience dataset *D.* For each time *t,* the DQN agent observes the state of the environment ***s***(*t*) for a given UE and, accordingly, it triggers an action ***a(t)*** based on an ε-Greedy policy that chooses actions according to the policy *π* in Equation (6) with probability 1− and a random action with probability *ε*. Then, the reward *r*(*t* + 1) is collected and the experience tuple < ***s***(*t*), ***a(t)***, *r*(*t* + 1), ***s***(*t* + 1) > is stored in the dataset. When the dataset is full (i.e., *l* experiences are stored), old experiences are removed from the dataset to save new ones. It is worth mentioning that during a number of InitialCollectSteps of the data collection, the actions are selected completely randomly by forcing *ε* = 1 in order to explore several states and start filling the dataset with experiences.

The process of updating the weights *θ* of the evaluation DNN is executed in every time window in parallel to the data collection and it makes use of the experiences stored in the experience dataset. Specifically, for each update a mini-batch *U*(*D*) of *J* past experiences is firstly selected randomly from the dataset. The selected experiences are denoted as *e_j_*, *j* = 1, …, *J*, and the components of tuple *e_j_* are denoted as < ***s_j_***,***a_j_***,*r_j_*,s_j_* >. Then, the update is performed based on the mini-batch gradient descent process. First, it computes the average mean squared error (MSE) loss *L*(*θ*) over all the *J* experiences in the mini-batch as:

(7) $$L\left(\theta \right)=\underset{e_{j}\in U\left(D\right)}{E}\left[\left(r_{j}+\tau .\underset{a^{\prime }}{max}\;Q\left(s_{j}{}^{*},a^{\prime },\theta^{-}\right)-Q\left(s_{j},a_{j},\theta \right){)}^{2}\right)\right]$$

Then, the mini-batch gradient descent of *L*(*θ*), denoted as ∇*L*(*θ*), is obtained by differentiating *L*(*θ*) with respect to *θ*, which yields:

(8) $$\nabla L\left(\theta \right)=\underset{e_{j}\in U\left(D\right)}{E}[(r_{j}+\tau \cdot \underset{a^{\prime }}{max}Q\left(s_{j}{}^{*},a^{\prime },\theta^{-}\right)-Q\left(s_{j},a_{j},\theta \right))\cdot \nabla_{\theta }Q\left(s_{j},a_{j},\theta \right)]$$

Then, the weights of the evaluation DNN *Q*(***s,a***,*θ*) are updated as:

(9) θ←θ+α·∇L(θ)

where *α* is the learning rate.

After each update of *θ*, the resulting *Q*(***s,a***,*θ*) is used for triggering new actions. Moreover, after *P* updates of *θ*, the weights of the target DNN are updated as *θ^-^* = *θ.*

The training operation of the DQN-agent associated to the *u*-th UE is summarized in Algorithm 1, which includes the data collection (lines 3–12) and the update of the weights *θ* of the evaluation DNN (lines 13–21 of Algorithm 1). The training duration in time steps is given by parameter MaxNumberOfTrainingSteps.
**Algorithm 1.** DQN training for the *u*-th UE1Initialize DNN counter *p* = 0.2**For** *t* = 0… MaxNumberOfTrainingSteps3     Collect state ***s*(*t*)** (see Section 3.2)4     Generate random *ε*’ (*ε*’ = 1 for the initial steps).5    **If** *ε*’<*ε*6     Choose randomly action
a(t)
**.**
7    
**Else**
8      Obtain action according to *π*.9    
**End if**
10   
Obtain reward *r*(*t* + 1) and *s*(*t* + 1) as a result of action
a(t)***(t)***. 11    **If** *D* is full (*l* samples are stored), remove the oldest one.12    Store experience < ***s***(***t***), ***a***(***t***), *r*(*t*+1), ***s***(***t***+**1**) > in *D*.
13     Randomly sample a minibatch of experiences *U*(*D*) from *D* of length *J.*14    
Compute the loss function
L(θ).15    
Compute the mini-batch gradient descent
∇L(θ).16    Update weights θ of evaluation DNN.17    **If**
*p* = *P*18      Update the weights of target DNN *θ^-^* = *θ* and set *p* = 0.19    
**Else**
20      *p* = *p* + 121    
**End if**
22 
**End for**


## 4. Results

This section evaluates the performance of the proposed solution by means of system level simulations. After describing the considered scenario for the training and evaluation as well as the relevant parameters for both stages in Section 4.1, the evolution of training process in order to obtain the policy is presented in Section 4.2. Section 4.3 describes the considered benchmarking strategies for assessing the performance of the proposed MC strategy. Then, Section 4.4, Section 4.5 and Section 4.6 provide the obtained performance results considering different situations, namely UEs following different trajectories, UEs in fixed positions and situations in which the number of MC-capable UEs is increased.

### 4.1. Scenario Description

**Figure 2:**
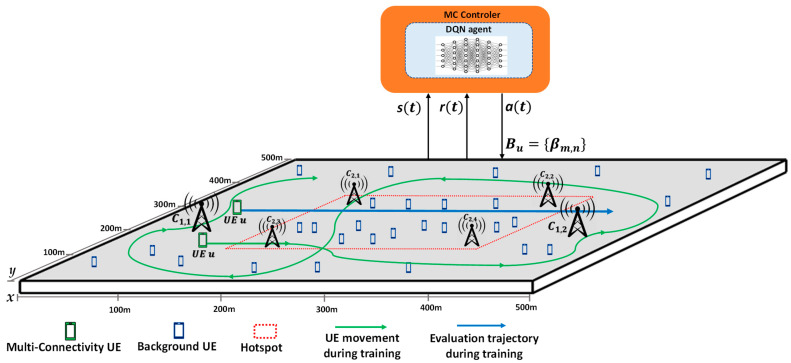
Graphic representation of the scenario used for training/evaluation.

The considered scenario is a square area of 500 m × 500 m, composed by four 5G NR cells and two LTE cells. As explained in Section 2.1, *C_m,n_* denotes the *n*-th cell of the *m*-th RAT, being *m =* 1 for LTE and *m =* 2 for 5G NR. Therefore, the LTE cells are identified as C_1,1_ and C_1,2_ and the 5G NR cells are C_2,1,_ C_2,2_, C_2,3,_ C_2,4_. The relevant parameters of the cells are presented in Table 1. Figure 2 illustrates the position of the different cells. The positions of the LTE and 5G NR cells were selected to illustrate a scenario in which 4 5G NR microcells are deployed in a denser area associated with a traffic hotspot, complementing the deployment of 2 LTE macrocells for wider coverage footprint.

The scenario assumes a non-homogeneous traffic distribution with UEs that support MC and other UEs that generate additional background traffic. The UEs that support MC follow specific trajectories moving at 1m/s along the scenario and have an active session during the whole simulation duration with a required bit rate *R_u_* = 50 Mb/s. These UEs can connect to up to $N_{max}=2$ cells from any of the two RATs.

The background traffic generation assumes Poisson session arrivals with aggregate generation rate 0.6 sessions/s and exponentially distributed session duration with average 120 s. As a result, the number of background UEs vary randomly during a simulation and the average number is 0.6·120 = 72 background UEs. A background UE remains static during a session. Fifty percent of the background UEs are randomly located inside a square hotspot of 250 m × 250 m centered at the middle of the scenario (see Figure 2). The rest of background UEs are randomly distributed in the whole scenario. Background UEs connect to the RAT/cell with the highest Signal to Interference and Noise Ratio (SINR). To capture the different bit rates achievable in the two technologies, when a background UE is connected to LTE, its serving cell allocates the needed resource blocks to achieve a bit rate of 2.5 Mb/s, and when it is connected to 5G NR, the allocation is to achieve a bit rate of 40 Mb/s.

The DQN model was developed in Python using the *TF-agents* library [32]. The DQN model parameters are detailed in Table 2. They were selected after conducting different tests of the algorithm with different configurations, then choosing a suitable configuration with satisfactory behavior in terms of, for example, reward performance, convergence, and stability. The presented results correspond to the performance obtained by the DQN algorithm with the MC configuration policy learnt by the DQN agent after a total of 1E6 policy updates according to the procedure of Section 3.2.

**Table 1:** Cell configuration parameters.

Cell Configuration Parameters		
Parameter	Value	
Type of RAT	LTE	5G NR
Cells position [x, y] m	[62, 250] [437, 250]	[187, 125] [187, 375] [312, 125] [312, 375]
Frequency	2100 MHz	26 GHz
Subcarrier separation	15 kHz	60 kHz
Nominal channel bandwidth	20 MHz	50 MHz
Number of available PRBs	100	66
Base station transmitted power	49 dBm	21 dBm
Base station antenna gain	5 dB	26 dB
Base station height	25 m	10 m
UE antenna gain	5 dB	10 dB
$\mathrm{Overload}\;\mathrm{threshold}\;\rho_{max}$	0.95	0.95
UE noise figure	9 dB	
UE height	1.5 m	
Path loss model	Urban Macrocell (UMa) model of Section 7.4 of [33]	Urban Microcell (UMi) model of Section 7.4 of [33]
$w_{max}$	95.04 MHz (corresponds to the case when MC is done with 2 cells of 5G NR)	

**Table 2:** DQN algorithm configuration parameters.

DQN Algorithm Parameters	
Parameter	Value
Initial collect steps	5000
MaxNumberOfTrainingSteps	1 × 10^6^
Experience Replay buffer maximum length (*l*)	1 × 10^5^
Mini-batch size (*J*)	256
DNN updating period (*P*)	2500 s
Discount factor (τ)	0.9
Learning rate (α)	0.0003
ɛ value (ɛ-Greedy)	0.1
DNN architecture	Input layer: 17 nodes Two hidden layers: 100 and 50 nodes Output layer: 58 nodes
Time window (Δ*T*)	1 s
Granularity Δ*β*	0.1

### 4.2. Training Evolution

The training process of the DQN algorithm is performed by considering a MC-capable UE moving along the scenario following trajectories according to random walk and with required bit rate *R_u_* = 50 Mb/s, while at the same time background UEs also generate traffic as explained previously. The DQN agent decides the MC connectivity configuration of the UE and, based on the obtained rewards, the decision-making policy is progressively updated as explained in Section 3.2. Figure 2 intends to graphically represent the scenario used for conducting the training and evaluation processes. At the beginning of the training the UE is located in the coordinates [X_1_ = 50, Y_1_ = 450]. Then, it moves with speed of 1 m/s following a random walk model in which it changes the direction (between ±$\frac{\pi }{4}$) with probability $p_{dir}=1/20$ at each time step. The green arrows in Figure 2 exemplify this process.

**Figure 3:**
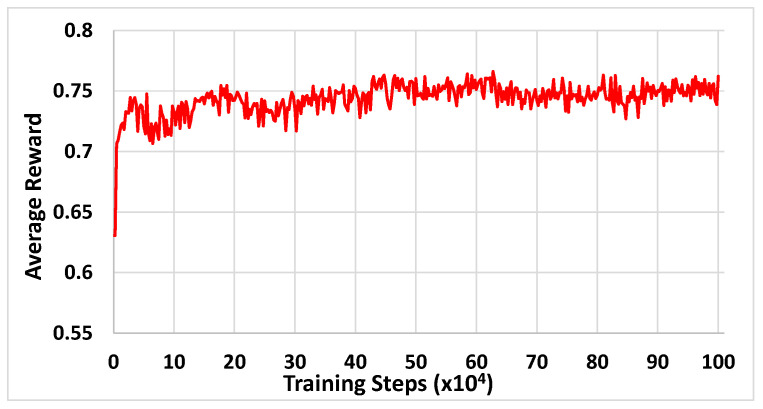
Evolution of the average reward as a function of the training steps.

The training is executed until reaching the maximum number of training steps Max Number Of Training Steps = 10^6^. To illustrate how the trained policy evolves when increasing the number of training steps, we obtained the learnt policy every 2500 training steps of the training process. Then, this policy was applied to an evaluation scenario in which an illustrative MC-capable UE follows the blue trajectory in Figure 2, starting from point [X_1_ = 50, Y_1_ = 300] and following a straight trajectory at 1 m/s up to the point [X_2_ = 450, Y_2_ = 300]. As a result, we measured the average reward obtained when applying the policy along this trajectory. Figure 3 presents the evolution of this average reward for the policies learnt every 2500 training steps.

The results show that at the beginning the average reward increases significantly, meaning that the training process is able to progressively learn better policies. Then, the reward tends to stabilize at around 40 × 10^4^ training steps, which gives an indication of the number of training steps needed to learn the policy. After this time, the reward fluctuations are only around 2%, which reflects the stability of the algorithm.

### 4.3. Benchmarking Strategies

Aiming to evaluate the performance of the policy obtained with our proposed DQN-based method against different strategies, two reference approaches were considered:

**Optimum strategy:** for a given UE, based on the set of candidate cells *A_u_* = {*C_m,n_*} and $N_{max}$ values, this strategy performs an exhaustive search process among all possible MC configurations at each time step and obtains the MC configuration that provides the highest reward. It is worth mentioning that it would not be a practical strategy for its implementation in real scenarios, mainly due to the large execution time for assessing all the possible MC configurations, so it is just considered as an upper bound of the DQN algorithm performance.

**SINR-based strategy**: in this strategy, at every time step all the traffic of the UE is served by the cell with the highest SINR value. This strategy reflects the classical approach of cellular systems in which the UE is served only by one cell, which is the one that provides the best quality (i.e., the highest SINR value). This criterion is considered for example in the specification [34] for the cell selection procedure, as well as in the measurement report triggering criteria of [35] to determine when one cell becomes better than another one, so that this can be used when deciding a handover. In addition, different works have also considered an SINR-based criterion for determining the cell a UE is connected to in a cellular system, such as [36,37]. It is worth mentioning that the SINR-based strategy can be implemented making use of handover procedures for changing the cell that the UE is connected to.

### 4.4. Performance Evaluation of the DQN-Based Strategy

#### 4.4.1. Performance for Different Trajectories

In order to assess the benefits brought by the proposed DQN-based MC strategy, this subsection compares the performance obtained by the proposed approach against the two reference strategies mentioned before, the *optimum strategy*, and the *SINR-based strategy*. To conduct this evaluation, the DQN-based MC strategy makes use of the policy learnt after completing the training process explained in Section 4.2. This policy is applied to the simulation of a UE of interest following 100 different straight trajectories of duration 400 s with different starting points and directions covering the entire evaluation scenario area. In each time window, the MC configuration according to the policy is applied and the resulting reward and throughput is measured. The same evaluation is executed when applying the two other reference strategies with the same trajectories.

**Figure 4:**
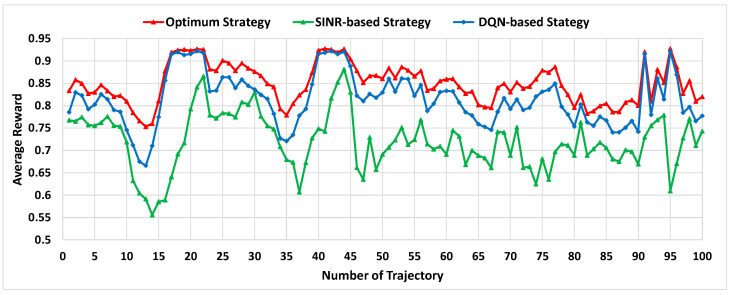
Average reward for different trajectories.

Figure 4 shows the average reward obtained for each one of the trajectories with all the considered strategies. It is observed that the DQN-based strategy achieves a performance very close to the optimum one in all the studied cases, with differences less than 1% in some of the trajectories, which confirms the good behavior of the proposed approach. In turn, if we analyze the reward of the 100 trajectories and obtain the average for all of them, the DQN-based approach outperforms the SINR-based approach in around 13.1% but the improvement can be as big as 50% in certain trajectories (e.g., trajectory 95). This main advantage of the DQN-based strategy with respect to the classical SINR-based strategy is due to smartly splitting the traffic of the UE among the cells to avoid overload and enhance the obtained bit rate. From an implementation perspective, while the SINR-based approach can be implemented relying on handover procedures, the DQN approach requires the support of the MC feature and that the base stations handling the involved cells are interconnected through the Xn interface in case of 5G NR or the X2 interface in case of LTE. Both requirements are now commonplace in live 4G/5G networks, which rely on the MC feature for supporting the widely used 5G NSA operation.

Regarding comparison with the optimum strategy, the error obtained with the DQN-based approach was computed as the percentage of difference between the reward of both strategies. The average of this error for all the trajectories was 4.32%. Similarly, the 5th percentile of the error was 0.35% and the 95th percentile was 7.94%. This reflects that the DQN-based approach achieves a performance quite close to the optimum but without having to exhaustively search for all the possible MC configurations.

**Figure 5:**
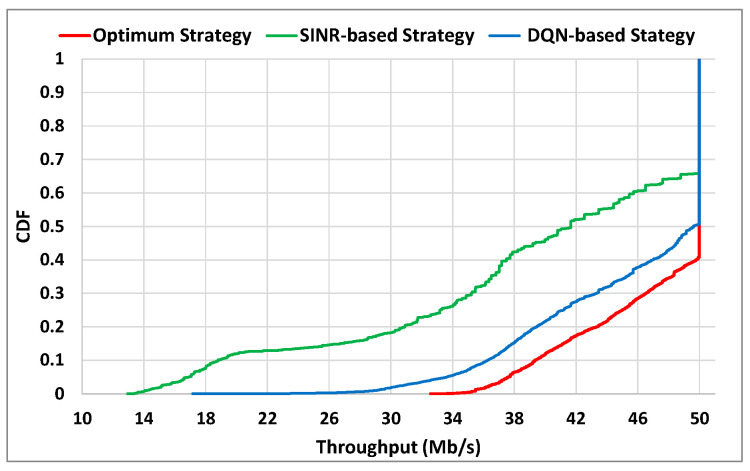
CDF of the throughput achieved by the UE of interest.

In order to assess the throughput performance, Figure 5 shows plots for each strategy of the cumulative distribution function (CDF) of the instantaneous throughput (*T_u_*) values obtained by the UE of interest with all the considered trajectories. Again, it is observed that the DQN-based strategy achieves a close performance to the optimum strategy and clearly outperforms the SINR-based strategy. In particular, with the DQN-based approach the UE throughput achieved 90% of the time is higher or equal to 37 Mb/s, while with the SINR-based approach this value is only 18 Mb/s. Similarly, the DQN-based approach is able to provide the throughput of 50 Mb/s during around 50% of the time, while the SINR-based approach only provides it 35% of the time.

**Figure 6:**
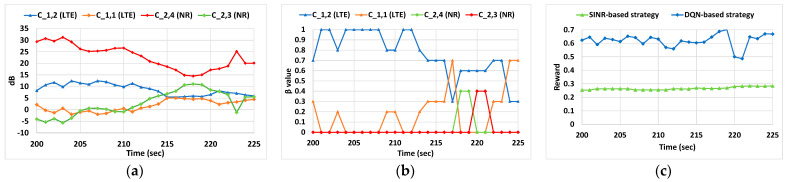
(**a**) SINR evolution of the LTE and NR cells in the analyzed period; (**b**) evolution of $\beta_{m,n}$ in the detected cells by the UE during the analyzed period and (**c**) reward in the analyzed period.

Aiming to further assess the behavior of the DQN-based strategy, we carried out a more detailed analysis focusing on a specific trajectory of a given UE starting in the point [X_1_ = 485, Y_1_ = 181] and following a straight trajectory at 1 m/s up to the point [X_2_ = 85, Y_2_ = 181]. For this specific trajectory, the SINR-based average reward was 0.634 while for the DQN approach this average reward rose to 0.835 which represents a gain of around 31%. Figure 6 presents a more specific and detailed analysis of this trajectory during a period of 25 s between the points (X_1_ = 285, Y_1_ = 181) and (X_2_ = 260, Y_2_ = 181). In particular, Figure 6a represents the evolution of the SINR experienced by the UE in the LTE and 5G NR cells during this period. Since one of the NR cells has a higher SINR value, this will be the selected cell with the SINR-based strategy during the whole analyzed period. In contrast, the DQN-based strategy is able to split the traffic through the LTE and 5G NR cells in accordance with the experienced conditions in terms of signal and load. In this case, Figure 6b shows the evolution of the values of *β_m,n_* selected by the algorithm. During the first 15 s the DQN-based strategy splits the traffic just using both LTE cells, i.e., *β*_1,2_ and *β*_1,1_ values are different from 0, even when those cells do not have the highest SINR values. For the remaining seconds, the traffic of the UE is delivered by using different MC configurations; for example, LTE-NR by adjusting the values of *β*_1,2_ and *β*_2,4_. As a result, Figure 6c shows the evolution of the reward with both strategies during the analyzed period, and it can be observed that the DQN-based strategy clearly outperforms the SINR-based one. In fact, if we consider average reward values for both strategies, the obtained with the SINR-based strategy is around 0.27, while the obtained with the DQN approach is 0.62, which is more than twice. It is worth mentioning that during the analyzed situation, the optimal strategy also provides a relatively low reward of 0.71. This is due to the traffic dynamics of the background UEs that lead to a high load in the cells during the analyzed time period. This high load does not allow ensuring the required bit rate with any of the combinations, which reduces the reward.

#### 4.4.2. Statistics on the MC Configuration

As explained previously, the goal of the proposed algorithm was to find the MC configuration that maximizes the reward under any experienced conditions by the UE. If we look at the evolution of $\beta_{m,n}$ in the detected cells by the UE during the analyzed period in Figure 6b we see that at some particular moments the DQN-based algorithm decided to send all the user traffic through a single cell. This means that during these moments, the algorithm decided that, based on the current state, not doing MC would result in a better performance, and this is confirmed through the reward observed in Figure 6c. Based on this, in the following we assess the decisions made by the algorithm in relation to when to apply MC and which are the technologies involved.

**Figure 7:**
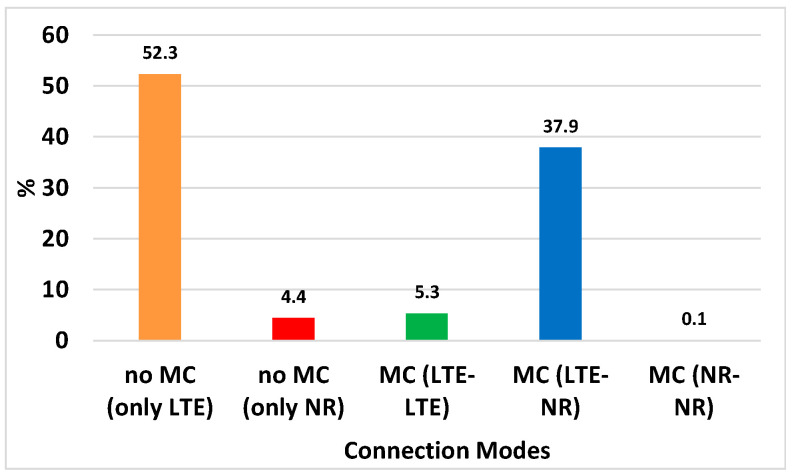
Statistics of connection modes for all moments of evaluation given 100 studied trajectories.

If we consider that the evaluation scenario has two LTE cells (C_1,1_ and C_1,2_) and four 5G NR cells (C_2,1,_ C_2,2_, C_2,3,_ C_2,4_), with $N_{max}=2,$ this means that the MC configuration can be done with five possible connection modes, namely *no MC with LTE; no MC with NR; MC with LTE-LTE; MC with LTE-NR and MC with NR-NR.* Figure 7 presents the percentage of time that the algorithm selected each one of these modes for the same 100 trajectories studied in Section 4.4.1. It is observed that during around 56% of the time the algorithm decided not to do MC and to connect instead to only one cell of LTE or one cell of 5G NR. In contrast, during the remaining 44% of the time it decided to do MC, particularly being the combination LTE-NR, the most used approach selected in around 38% of the time. This is logic given that LTE cells have a bigger coverage area than the NR cells, but these have more bandwidth. Therefore, this connection mode could be helpful at the time of fulfilling; for example, the UE data rate requirements while the UE is moving. Given the statistics presented in Figure 7 and after observing previously that the DQN-based solution performs much better that the SINR-based one, the importance of deciding the correct connection mode and the multi-connectivity configuration when required is clear.

### 4.5. Performance for Different Fixed Positions

**Figure 8:**
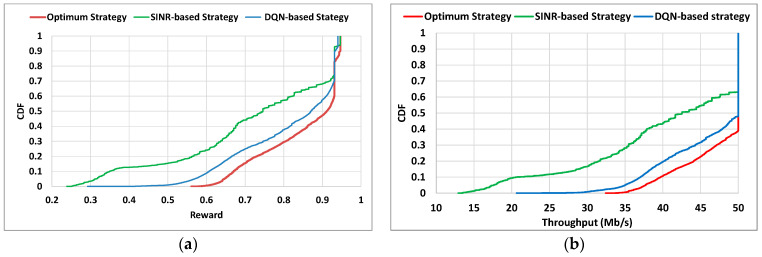
(**a**) CDF of average reward for the UE of evaluation; (**b**) CDF of throughput of the evaluated UE.

In the context of 5G, it is also pertinent to analyze situations in which the users/devices remain stationary at fixed positions, since some services have this characteristic (e.g., Fixed Wireless Access, smart cities with sensors or cameras at lamp posts). We carried out an evaluation performance of the DQN-based strategy considering the same scenario explained in Section 4.1 but this time the evaluation UE remained in a fixed position. In order to explore a variety of situations, we studied the performance of the UE for 400 s in 19 × 19 = 361 different positions all around the 500 × 500 m scenario, selected from a grid of locations in steps of 25m in both horizontal and vertical directions. We compared the results against the optimal and SINR-based strategies. Figure 8 shows the CDF of the instantaneous values of throughput (*T_u_*) and reward for all the considered positions. It is shown that, like in the evaluation with trajectories, the performance for fixed positions is close to the optimum one and clearly outperforms the SINR-based strategy. In fact, when analyzing some particular fixed positions, the DQN-based strategy is able to outperform the reward of the SINR-based one in up to 65%.

**Figure 9:**
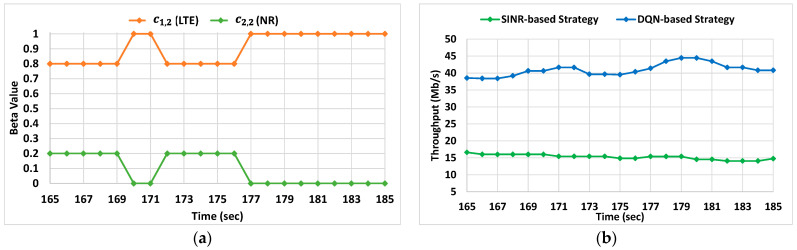
(**a**) Evolution of $\beta_{m,n}$ in the analyzed period; (**b**) obtained throughput in the analyzed period.

Aiming to explain more in detail the situations where the DQN-based strategy can come up with more different decisions than the SINR-based approach, we focused on a specific period of time of one of the evaluated fixed positions where the UE was located in the coordinates (X = 300, Y = 350). For this position, the UE experienced SINR values in the 5G NR (C_2,2_) and LTE (C_1,2_) cells equal to 45.7 dB and 33.8 dB respectively. Due to these perceived values, the SINR-based strategy selected the 5G NR cell during the entire period. In contrast, as shown in Figure 9a, the DQN-based strategy considered the current cell loads, and decided to split the traffic among the two cells. In the case of fixed positions, splitting the traffic becomes important because, if the higher-SINR-detected cell is serving other users, at some point it can get overloaded. However, by considering other aspects such as load as the DQN-based strategy does, it is possible to avoid this type of issue while improving the throughput obtained by the UE/device of interest. This effect can be seen clearly in Figure 9b. For the 20-s analyzed period the obtained average throughput with the SINR-based strategy is 15.3 Mb/s while by using our proposed approach for the same period, the average throughput reaches 40.72 Mb/s, which represents a gain of around 166%. This result reinforces the idea about the importance of optimizing the MC configuration of the UEs.

Considering the results for all the different fixed positions, it was seen that the error of the DQN-based approach with respect to the optimum was, on average, 3.76%, the 5th percentile was 0.08% and the 95th percentile was 13.3%. Similarly, considering all the results obtained with the 100 trajectories of Section 4.4 and the fixed positions of Section 4.5, the average error was 3.9% and the distribution provides a 5th percentile of 0.09% and a 95th percentile of 12.5%. This reflects the close to optimum behavior obtained with the proposed approach.

### 4.6. Performance Evaluation in Scenarios with Multiple MC-Capable UEs

This section considers the performance of the DQN-based solution when there are multiple MC-capable UEs coexisting in the same evaluation environment and applying the learnt DQN-based policy.

The study consisted of five different simulations, each one with a distinct number of MC-capable UEs, ranging from 5 to 25, all of them with a *R_u_* = 25 Mb/s. During the simulation time each UE moved following a different trajectory and generated traffic during the whole evaluation time, equal to 400 s. The background traffic had a generation rate of 0.2 sessions/s and an exponentially distributed session duration with an average 120s, resulting in an average of 24 background UEs during a simulation.

**Figure 10:**
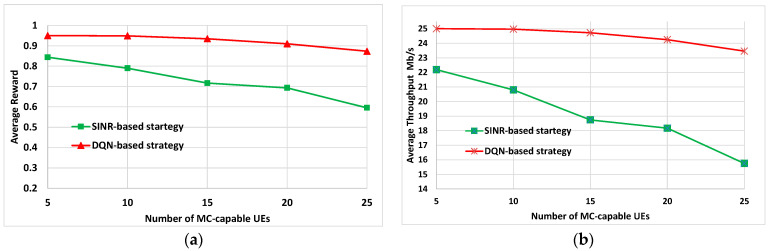
(**a**) Average reward values as a function of the number of MC-capable UEs; (**b**) average throughput values as a function of the number of MC-capable UEs.

The results presented in Figure 10 were obtained by averaging the performance for the different UEs in each evaluation with the SINR-based strategy and with the proposed DQN-based strategy. Figure 10a depicts the performance of our solution in terms of reward. It can be seen that the gain of DQN-based strategy with respect to SINR-based strategy increases from 11.2% for 5 UEs up to 31.7% for 25 UEs.

Regarding the throughput performance, a similar assessment was done and the results are presented in Figure 10b. They show that the proposed method outperforms the SINR-based approach for all the considered numbers of users., with a gain of around 33% for the case with 25 UEs. Notice how by applying our solution it tends to keep the obtained throughput very close to the requirement of *R_u_* = 25 Mb/s.

## 5. Conclusions and Future Work

This paper proposes the use of Deep Q-Network for splitting the traffic of a UE among cells when using multi-connectivity depending on the current traffic and radio conditions experienced by the UE in the involved cells. The proposed strategy was evaluated and compared against the optimum case and against a classical SINR-based approach in different evaluation scenarios, involving UEs following trajectories and stationary UEs. Results show the capability of the DQN agent to learn a quasi-optimal policy, providing a reward that on average is only 3.9% lower than the reward of the optimum strategy. It was also seen that the DQN-based approach clearly outperforms the SINR-based approach with reward differences that can be up to 50% for certain trajectories. In turn, for the case of fixed positions, results show that the DQN-based strategy can achieve throughput gains of up to 166%with respect to the SINR-based strategy at certain times. The statistics regarding the different connection modes used by the DQN strategy confirm the capability of the algorithm to optimize the decision on when to use MC or not, to maximize the performance. Moreover, a performance analysis of the DQN-based strategy was conducted when applied to different numbers of MC-capable users coexisting in the same evaluation environment. The results from this study reveal a significant better performance for all the MC-capable users when applying the solution, which reinforces and confirms the relevance of optimizing the MC configuration.

Overall, our results reflect a promising performance of the proposed DQN-based approach that opens the door for continuing the work around some future research lines. Particularly, the solution could be extended to incorporate other types of traffic with service requirements other than the bit rate considered here. For example, Ultra Reliable and Low Latency Communications (URLLC) services can be considered to exploit MC for enhancing reliability, e.g., by duplicating packets through multiple cells. The exploitation of the proposed mechanisms in other scenarios in which MC can be used with other UEs acting as relays can also be tackled as future work.

## Data Availability

Not applicable.
